# From genes to traits: maximizing phosphorus utilization efficiency in crop plants

**DOI:** 10.3389/fpls.2025.1527547

**Published:** 2025-04-08

**Authors:** Sumer Zulfiqar, Ran Gu, Yan Liu, Yaowei Zhang

**Affiliations:** ^1^ College of Horticulture & Landscape Architecture, Northeast Agricultural University, Harbin, China; ^2^ Key Laboratory of Biology and Genetic Improvement of Horticultural Crops (Northeast Region), Ministry of Agriculture and Rural Affairs, Harbin, China

**Keywords:** phosphorus use-efficiency, phosphate signaling, phosphate homeostasis, arbuscular mycorrhizal symbiosis, phosphate transporters

## Abstract

Phosphorus (P) is a critical macronutrient for plant growth, but its limited availability requires efficient utilization strategies. The excessive use of P fertilizers leads to low phosphorus utilization efficiency (PUE), causing severe environmental impacts and speeding up the exhaustion of P mineral reserves. Plants respond to inorganic phosphate (Pi) deficiency through complex signaling pathways that trigger changes in gene expression, root architecture, and metabolic pathways to enhance P acquisition and utilization efficiency. By exploring the interplay between genetic regulators and microorganisms, cultivars with superior PUE traits can be developed, which will ensure agricultural resilience and productivity in the face of depleting global P reserves. We highlight the synergistic interaction between genetic regulators and microorganisms to boost PUE as well as recent advancements in unraveling molecular mechanisms governing P homeostasis in plants, emphasizing the urgency to improve plant traits for improved P utilization.

## 1 Introduction

Phosphorus (P) is a vital macronutrient essential for plant growth and reproduction. However, the complex physiochemical reactions in soil often lead to the fixation and precipitation of P in forms that are insoluble and less available for plant uptake such as calcium phosphate (Ca-P), aluminum/iron phosphate (Al/Fe-P), and calcium/magnesium phosphate (Ca/Mg-P) (López-Bucio et al., 2002; Malhotra et al., 2018). This limitation in P availability poses a significant challenge for sustainable agriculture and global food security. In order to overcome these limitations, P-containing fertilizers are typically employed in agriculture systems. Unfortunately, only a small amount of the applied P is taken up by plants immediately. The remaining runoff into water bodies, contributing to eutrophication and harmful algal blooms (Zak et al., 2018). Additionally, P in mineral fertilizers is predominantly derived from phosphate rock, a finite resource. Therefore, the development of P-efficient cultivars that maximize phosphorus utilization efficiency (PUE)-the ratio of the amount of P taken up by the plant to the amount of P supplied in the environment-is paramount for enhancing crop yields while reducing the need for fertilizer inputs. This approach is crucial for conserving global P resources and mitigating environmental issues.

The regulation of PUE is a complex network of genes, proteins, and metabolites, including various signaling pathways such as phosphate starvation response (PHR) and root development pathways (Baker et al., 2015). Key players in this network include the PHT1 phosphate transporters, PHO1, PHR1, SPX domain-containing proteins, and IPS1 (Induced by Phosphate Starvation 1). Central to the regulation of PUE is the PHR pathway, which activates the expression of phosphate starvation-induced (PSI) genes under Low-Pi conditions (Roychowdhury et al., 2023). PHR transcription factors bind to PHR1-binding sequences (P1BS) in the promoters of PSI genes, facilitating the expression of genes essential for Pi-uptake, remobilization, and metabolic adjustments (Abdullah et al., 2024). In parallel, root development pathways significantly enhance PUE by improving root system architecture (RSA); they promote increased root density, longer root hairs, and more lateral root branches, all of which enhance the plant’s ability to absorb Pi from the topsoil (Sun et al., 2018). For instance, auxin signaling plays a critical role in lateral root initiation and elongation in response to Pi deficiency, expanding the root surface area available for nutrient absorption (Zhang et al., 2023a). Additionally, P deficiency triggers the exudation of protons, organic anions (such as citrate and malate), and acid phosphatases, which collectively solubilize fixed P in the soil, making it more accessible (Roychowdhury et al., 2023). These genetic and physiological adaptations also foster beneficial interactions with microorganisms, like arbuscular mycorrhizal fungi that expand the soil volume utilize for P uptake and phosphate-solubilizing bacteria, that elevate soil P availability P availability (Pang et al., 2018). By introducing these genes through molecular breeding into local cultivars, plant characteristics can be enhanced.

To increase PUE under Low-Pi conditions, two approaches should be utilized: (i) genetic enhancement and (ii) soil enzyme manipulation. Genetic enhancement can lead to higher yields with lower P input, reducing environmental impacts from excessive fertilizer usage. For example, overexpression of phosphate transporters, such as *PHO1;2* in rice can lead to a reduction of Pi accumulation in grains and increased grain yield and PUE (Ma et al., 2021). Under normal Pi conditions, *PHO1;2* helps maintain adequate phosphate homeostasis, ensuring that plants have sufficient nutrients for growth. In contrast, during Low-Pi conditions, *PHO1;2* expression is upregulated to enhance phosphate uptake and distribution within the plant. Additionally, the manipulation of enzymes that mediate Pi mobilization from organic P pools plays a critical role in enhancing PUE in crops. Enzymes such as phosphatase and phytase are known to facilitate the conversion of organic P into forms that plants can absorb, thereby enhancing PUE. Phosphatases hydrolyze phosphate esters, which are prevalent in organic matter, releasing Pi that plants can utilize. Phytase, specifically, breaks down phytate, a form of organic P found in seeds and other plant materials, into free Pi and myo-inositol, thus facilitating its availability to plants. For instance, overexpressing glycerophosphodiester phosphodiesterases (GDPD2) has been shown to enhance Pi remobilization from membrane phospholipids and increase tiller number in rice (Mehra et al., 2019). Understanding the molecular mechanisms behind these genes and regulatory pathway is crucial for developing P efficient cultivars that improve PUE. In addition, advancements in understanding the remobilization of liberated Pi within plants, including various P fluxes and cell-specific P allocation, also offer promising insights for enhancing PUE in crops. This knowledge contributes to optimizing P fertilization practices, enhancing crop yields, and mitigating the environmental impacts associated with excessive P application. Previous studies have explored mechanisms of P transport and signaling in plants, as well as methods for improving PUE (Zhang et al., 2014; Wang et al., 2021a; Han et al., 2022). Despite these advancements, the regulation of P by genetic regulators in conjunction with soil enzymes to enhance PUE in plants has not been previously reported. In this review, we present a novel approach that integrates genetic regulators and soil enzymes to enhance PUE in plants, offering a new perspective for additional research in this field. Our investigation of the physiological and traits for optimized P utilization.

## 2 Phosphorus homeostasis in plants: mechanisms of uptake, transport, and storage

Plants have evolved a sophisticated system of transporters to ensure efficient absorption of Pi from the soil and its redistribution within the plant. Key transporters like PHOSPHATE TRANSPORTER 1 (PHT1) proteins, such as AtPHT1;1, AtPHT1;4 in Arabidopsis and OsPHT1;2, OsPHT1;3, OsPHT1;4 in rice, play a crucial role in Pi uptake and transport (Victor Roch et al., 2019; Wang et al., 2021a). Recent studies have shown that in wheat, the overexpression of *TaPHT1;9* significantly enhances Pi uptake and PUE (Wang et al., 2021). Similarly, in soybean and barley, the overexpression of *GmPT1* and *HvPHT1;1*, respectively, has been associated with improved Pi uptake and increased translocation to the shoots (El Ifa et al., 2024; Yang et al., 2024). The Casparian band aids in loading Pi into xylem vessels for transportation to shoots, while PHOSPHATE 1 (PHO1) proteins mediate Pi export from cells near the xylem (Poirier et al., 1991). Furthermore, Pi redistribution from senescing tissues to developing organs involves various PHT1 and PHO1 proteins (e.g., AtPHT1;5 in Arabidopsis, ZmPHT1;7 in maize, and OsPHT1;1, OsPHT1;2, OsPHT1;3 in rice). Vacuolar Pi transporters, including Vacuolar Phosphate Transporter 1 (AtVPT1), are responsible for storing excess Pi in vacuoles (Liu et al., 2015). This storage mechanism is finely regulated by the expression of PHT and PHO1 genes, influenced by Pi deficiency and excess Pi supply (Han et al., 2022). Transcription factors such as PHRs and WRKYs play a crucial role in this regulation. Additionally, the phosphorylation and dephosphorylation of Pi transporters are modulated by various proteins like PHOSPHATE TRANSPORTER TRAFFIC FACILITATOR 1 (AtPHF1), *Arabidopsis* apoptosis‐linked‐gene 2 (ALG2), interacting protein X (AtALIX), and nitrogen limitation adaptation (AtNLA) (For details, see recent review Wang et al., 2021 (Wang et al., 2021a)).

**Figure 1:**
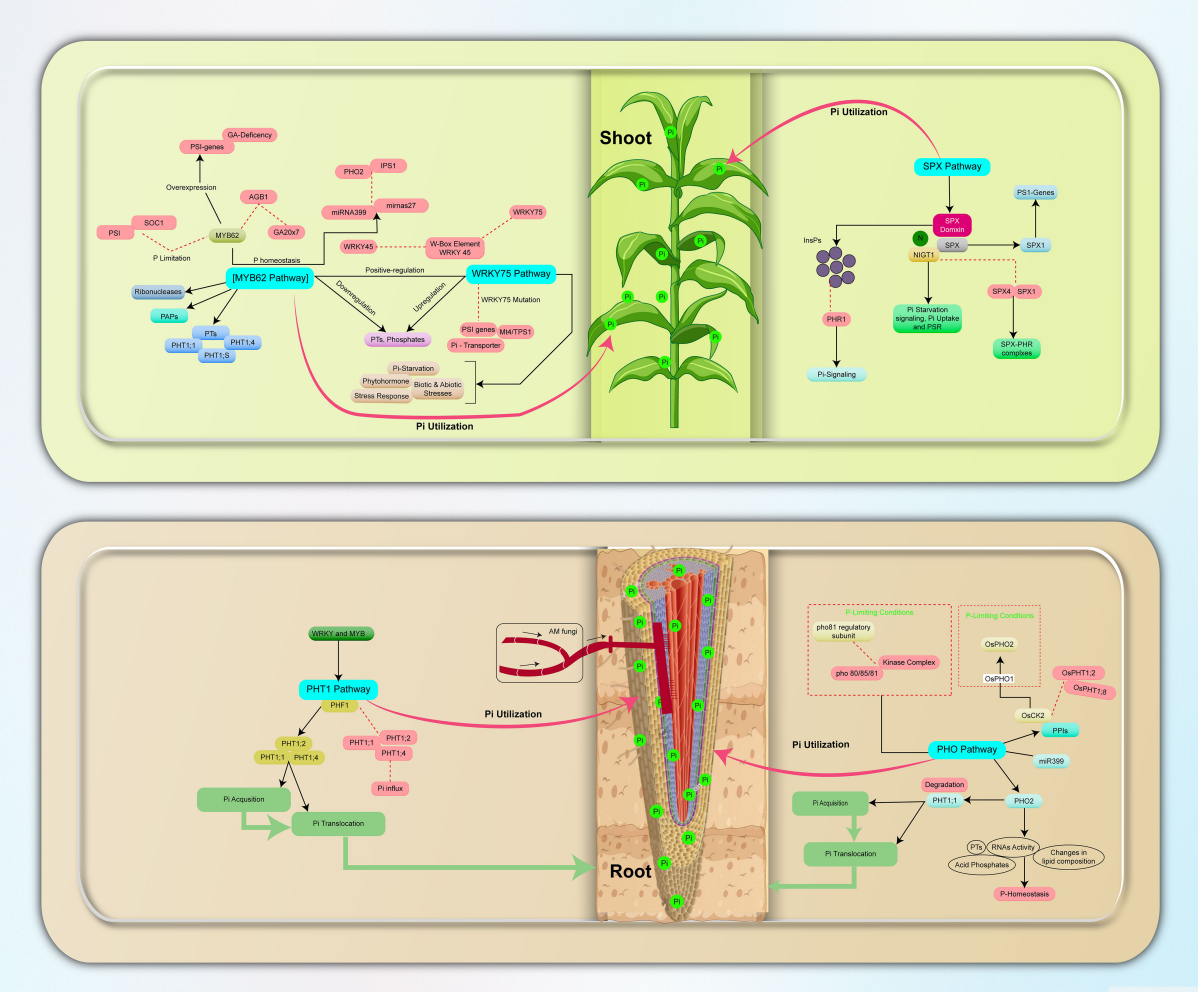
Overview of phosphate signaling pathways in plants; Pi acquisition, utilization, and translocation. The PHO Pathway regulates Pi-responsive genes and increases Pi uptake, the PHT1 Pathway mediates high-affinity Pi transporters and membrane localization, The PHT1 pathway plays a crucial role in mediating the symbiotic relationship between AMF and plants, especially in nutrient-deficient soils. While AMF are known to enhance nutrient uptake, the specific regulatory mechanisms of PTs under combined abiotic stress conditions remain poorly understood. MYB62 and WRKY75 Pathways act as transcription factors in Pi Homeostasis and Stress Response, while the SPX Pathway negatively regulates Phosphate Transport and Signal Transduction to ensure effective Pi regulation in plant systems. PP-InsPs promote the interaction between SPX proteins and the PHR1 transcription factor, leading to PHR1 inactivation. Changes in PP-InsP levels in response to Pi deficiency may facilitate plant adaptation to stress by modulating the activity of SPX-containing proteins and their interactors. Blockage and inhibition are indicated by dotted lines and red boxes, respectively.

**Figure 2:**
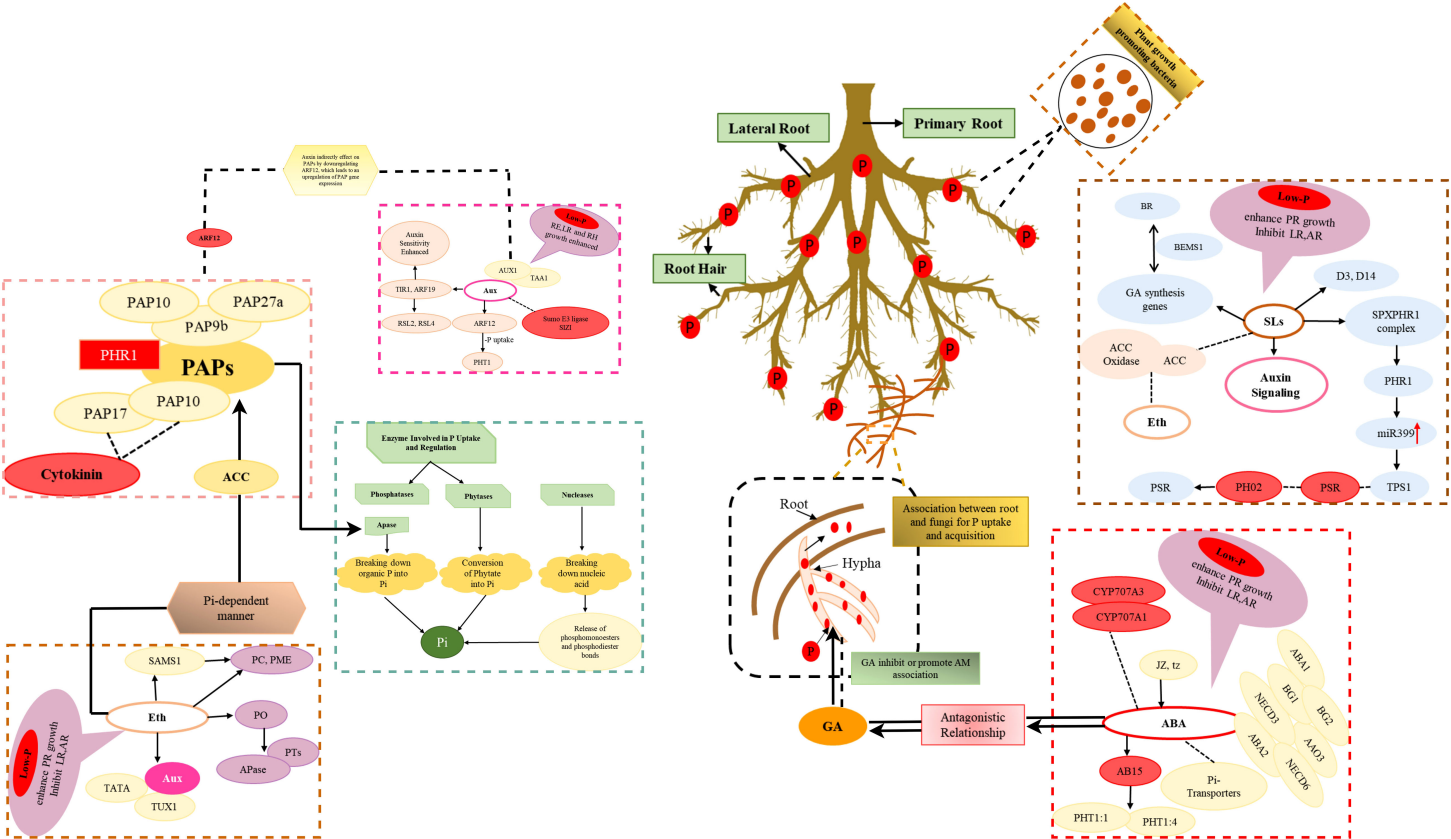
Physiological mechanisms of phosphorus utilization regulated by hormones, enzymes and microorganisms. dotted lines inside the box indicate negative regulation, while arrows indicate positive regulation. Aux, Auxin; Eth, Ethylene; SLs, Strigolactones; ABA, Abscisic acid; PGPB, Plant Growth-Promoting Bacteria.

Under Pi-sufficient conditions, PHT1 proteins facilitate Pi uptake at the plasma membrane, while vacuolar influx transporters like VPT1/PHT5;1 store Pi in vacuoles (Luan and Lan, 2019). Conversely, under Pi-deficient conditions, regulatory mechanisms involving PHRs, WRKYs, and proteins like PHO2 and PP95 enhance the induction of PHT1 transporter genes to improve Pi acquisition (Wang et al., 2021c). This intricate balance of regulatory mechanisms ensures the proper uptake, transport, and storage of Pi within plant cells to maintain Pi homeostasis (**Figure 1**). Efficient P utilization in plants relies on various other complex physiological processes involving phytohormones and enzymes (**Figure 2**). This orchestration is essential for optimizing Pi utilization in plants, ultimately influencing crop yields and productivity. To further enhance plant growth and productivity, it is crucial to understand and develop effective strategies for improving P utilization.

## 3 Genetic regulators influencing PUE

### 3.1 Decoding phosphate signaling pathways in plants

Plant P signaling pathways are crucial for growth and respond to changing environmental conditions. The PHO, PHT1, MYB62, WRKY75, and SPX pathways play key roles in sensing and responding to P availability (**Figure 1**).

#### 3.1.1 PHO pathway: regulation of Pi-responsive genes and increased phosphate uptake

The PHO pathway is a crucial signaling pathway in plants it allows the plants to sense and respond to changes in P availability, the PHO pathway ensures optimal growth and survival. This pathway controls P uptake through a complex signaling cascade. The central component of PHO pathway is the PHO1 protein, which facilitates the transfer of Pi from roots to shoots. Studies have highlighted the crucial functions of specific isoforms of PHO1, such as *OsPHO1;2* in rice (Ma et al., 2017) and *ZmPHO1;2* in maize (Ma et al., 2021), in Pi redistribution and homeostasis within seeds (Vogiatzaki et al., 2017). Another important protein in the PHO pathway is PHO2, which acts as a transcription factor and regulates the expression of multiple genes involved in P uptake and homeostasis. PHO2 is an E2 ubiquitin conjugase that controls phosphorus starvation-responsive genes in plants. It is post-transcriptionally regulated by miRNAs like miR399, while its downstream targets include Phosphate transporters (PTs), RNase activity, acid phosphatase, and alterations in lipid composition (**Figure 1**), all of which contribute to managing P homeostasis in plants (Prathap et al., 2022). The pathway also includes important regulatory layers involving kinases and phosphatases. For instance, the Pho80/85/81 kinase complex is inactivated by the regulatory subunit Pho81 when P becomes limiting (Schneider et al., 1994; Austin and Mayer, 2020). The kinase subunit Pho85 phosphorylates the transcription factor Pho4, promoting its cytosolic localization and inhibiting the expression of genes controlled by the PHO pathway (**Figure 1**). Moreover, the PHO pathway is intricately regulated at both the transcriptional and post-transcriptional levels, with key regulatory factors such as PHO2 and miRNAs (Prathap et al., 2022). For example, in hexaploid wheat, three homologous PHO2 genes—*TaPHO2-A1, -B1*, and *-D1*—have been identified, all containing miR399-binding sites. Knockout of these genes results in increased leaf P concentrations, with *TaPHO2-D1* showing the most significant impact on plant growth. Notably, disabling *TaPHO2-A1* enhances P uptake and grain yield under Low-P conditions without adversely affecting yields in high P environments (Ouyang et al., 2016), This evidence demonstrated the potential of targeting these genes to improve wheat yields while reducing reliance on fertilizers. Environmental influences, including sulfate availability and sugar levels, also play a significant role in modulating the activity of the PHO pathway. Additionally, the complexity of this pathway is further illustrated by protein-protein interactions (PPIs) that occur within the PHO pathway. For example, in rice the PROTEIN PHOSPHATASE 95 (OsPP95), dephosphorylates *OsPHT1;2* and *OsPHT1;8* in an antagonistically manner with respect to OsCK2, this interaction helps to regulate the phosphorylation status and trafficking of these transporters. *OsPHO2* mediates the degradation of OsPP95 under Pi-sufficient conditions (Yang et al., 2020). OsCK2 affects the stability of *OsPHO2* through phosphorylation, thereby influencing Pi homeostasis through its target protein *OsPHO1* (Dai et al., 2022). In Arabidopsis, PHO2 directly interacts with PHT1;1 at the endoplasmic reticulum (ER) membrane, controlling its ubiquitination and subsequent degradation (**Figure 1**), corroborating its role in regulating Pi uptake (Huang et al., 2013).

Recent advances have revealed that the pathway is interconnected with various nutritional and hormonal signaling cascades, including strigolactones (SLs) and brassinosteroids (BR), enhancing the plant’s ability to adapt to varying nutrient conditions (Yang et al., 2023; Puga et al., 2024). Notably, these crosstalk mechanisms facilitate the balance between growth and nutrient acquisition, reinforcing that the PHO pathway is not merely a linear response system but rather an integral node linking plant developmental processes with nutrient signaling. Thus, the PHO pathway exemplifies a sophisticated network that manages Pi homeostasis through an elaborate integration of signaling inputs, enabling plants to adapt effectively to Pi scarcity and ensuring their survival and productivity in varying environmental conditions.

#### 3.1.2 PHT1 pathway: high-affinity phosphate transporters and membrane localization

The PHT1 pathway is also a crucial mechanism in plants for the uptake of Pi from the soil, which is essential for various cellular processes such as energy metabolism and nucleic acid synthesis (**Table 1**).

**Table 1:** Functional characterization of PHT genes.

Gene Family	Member Genes	Function	Functional Role and Characterization	Method of Characterization	Plant Species	Reference
Phosphate Transporters (PHTs)	*LaPHT1, LaPHT2, LaPHT3, LaPHT4*	P-absorption	Identified 35 PHT genes distributed across 16 chromosomes. *LaPHT1* subfamily showed increased expression under P-deficient conditions, aiding in P absorption and adaptation.	Transcriptomic and qRT-qPCR	White lupin	(Aslam et al., 2022)
	*TaPHT1.1, TaPHT1.2, TaPHT1.4, TaPHT3.1, TaPHT4.2*	P-uptake during grain development	*TaPHT1.1* enhances P uptake in aleurone tissues, contributes to higher P accumulation in mature seeds. Upregulated *TaPHT1.2* plays a critical role in downregulating under P-deficient conditions. *TaPHT1.4* and *TaPHT3.1* Significant in P accumulation and remobilization during grain filling; strong correlation with grain yield under low P conditions. *TaPHT4.2* Critical for Pi transport in the embryo during germination; facilitates seedling establishment by mobilizing P reserves. Functions in Pi transport within endosperm tissues; enhances overall grain P content, impacting grain quality and yield.	qRT-PCR	Wheat	(Shukla et al., 2016)
PHT1	*HvPHT1;1, HvPHT1;5, HvPHT1;11, HvPHT1;12*	P-uptake enhancement	*HvPHT1;1* Enhances Pi uptake during P starvation; *HvPHT1;5* induced under sucrose deficiency; *HvPHT1;11* facilitates Pi uptake through AM fungi association. *HvPHT1;12* Involved in enhancement of P uptake via AM fungal colonization	qRT-PCR	Barley	(Srivastava et al., 2021)
	*OsPht1;1, OsPHT1;7*	Increased P content, Mediates Pi transport	Overexpression of *OsPht1;1* increases P content; Involved in Pi redistribution; higher expression in anthers; affects germination and seed-setting rate.	Semi-quantitative RT-PCR and Yeast complementation, *Xenopus laevis* oocyte assay, reverse-transcription quantitative polymerase chain reaction, histochemical analyses, immunostaining.	Rice	(Sun et al., 2012) (Dai et al., 2022)
UGPase	Ugp1	Sucrose accumulation	*Ugp1* is a UDP-glucose pyrophosphorylase that enhances sucrose accumulation during Pi starvation stress, affecting P signaling. Overexpression leads to increased sucrose and Pi accumulation while regulating expression of PHT1 genes.	Over-expression	Rice	(Zhang et al., 2023b)
	*OsPht1;6 (OsPT6)*	Growth and biomass	Overexpressing *OsPT6* in rice improves growth and P accumulation. Higher Pi uptake leads to increased grain yield.	Over-expression	Rice	(Zhang et al., 2014)
	*LpPHT1;4, LpPHT1;1*	Pi-uptake	Expression increased under P starvation; can complement yeast mutant Δpho84 under Pi-deficient conditions. Not correlated with P supply.	qRT-PCR and yeast complementation assays	Ryegrass	(Parra-Almuna et al., 2020)
	*GmPHT1;1*	AMF colonization	Enhances Pi transfer to roots through AM fungi interaction.	qRT-PCR	soybean	(Fan et al., 2013)
	*TaPHT1;9*	Pi-absorption	Mutants showed reduced PUE under low Pi; transgenic rice highlighted enhanced PUE.	CRISPR-edited *TaPHT1;9* wheat mutants	Gene *TaPHT1;9* is studied in both wheat and rice to understand its effects on PUE	(Chen et al., 2024)
	*TaPHT1;9-4B*	Pi-acquisition	Regulates *TaPHT1;9-4B*, essential for Pi acquisition; transgenic expression improves Pi uptake in rice.	VIGS, CRISPR/Cas9	Wheat	(Wang et al., 2021)
	PHT1;2, PHT1;3	Pi-uptake	Constitutive expression across millets, notable induction under Low-P conditions. Involved in Pi distribution and remobilization.	qRT-PCR	Millets	(Maharajan et al., 2019)
	*BnPht1;4*	High-affinity phosphate transporter	*BnPht1;4* is upregulated under Pi deficiency and has impaired activity at root tips.	Quantitative reverse-transcription PCR analysis, promoter activity assay, cis-element analysis, yeast one-hybrid assay	Rapeseed, Arabidopsis	(Ren et al., 2014)
PHT2	*GmPHT2;1, GmPHT2;2*	P-metabolic homeostasis	Up-regulated under Low-Pi conditions; *GmPHT2;2* exhibits significantly higher expression under Low-Pi stress compared to *GmPHT2;1.*	qRT-PCR	Soybean	(Wei et al., 2023)
	*TaPHT2;1*	Chloroplast Pi levels	Knocking down reduces chloroplast Pi levels, decreasing photosynthesis; role in Pi signaling and regulation of PHTs.	Semi-quantitative RT-PCR and qPCR analyses	Wheat	(Guo et al., 2013)
PHT3	PHT3;1	Mitochondrial Pi transport	Ubiquitously expressed; transports Pi from cytosol into the mitochondrial matrix, supporting ATP synthesis.	Over-expression	Arabidopsis	(Jia et al., 2015)
PHT4	PHT4;1, PHT4;4	Pi transport in chloroplasts	PHT4;1 enhances photosynthesis and P utilization, PHT4;4 supports carbohydrate synthesis.	qRT-PCR	Arabidopsis	(Guo et al., 2008)
	PHT4;1, PHT4;3, PHT4;5, PHT4;6	Various Pi transport roles	Involved in H+/Na+-coupled Pi transport; PHT4;3 and PHT4;5 localize to chloroplasts; PHT4;6 involved in Golgi Pi export.	Heterologous expression in yeast, Pi transport assays, subcellular localization using GFP fusions, quantitative RT-PCR for expression analysis.	Arabidopsis	(Versaw and Garcia, 2017)
PHT5	Vacuolar phosphate transport (VPT1) (PHT5;1), PHT5;2, PHT5;3	Tonoplast influx transporter	Functions as a tonoplast influx transporter for Pi storage; important for maintaining cytosolic Pi homeostasis under Pi starvation.	GUS-promoter agrobacterium transformation and qRT-PCR	Arabidopsis, rice	(Liu et al., 2015); (Liu et al., 2016)
	*BnA09PHT5;1b, BnCnPHT5;1b*	Cellular Pi homeostasis	Crucial for cellular Pi homeostasis; double mutants exhibit growth deficiencies and higher cellular Pi, affecting seed yield and traits.	Functional analysis in yeast to assess Pi transport activity and characterization of double mutants using CRISPR-Cas9 to evaluate their impact on growth and Pi accumulation.	Rapeseed	(Han et al., 2022)

The pathway primarily involves high-affinity PHT1, which are integral membrane proteins responsible for the uptake and transport of Pi across cell membranes (Chien et al., 2022).

The PHT1 transporters are found in the plasma membrane of root cells (specifically in the root hair and the epidermal cells of the root) (Wang et al., 2021c). The PHT pathway begins with the detection of Pi levels in the soil by root cells. This sensing mechanism activates a cascade of responses, including the activation of PHRs, which upregulate the transcription of PHT1 transporters, such as *PHT1;1*, enhancing Pi uptake (Ayadi et al., 2015). Additionally, PHRs increase the expression of *microRNA827 (miR827)*, which targets and degrades NLA, a ubiquitin E3 ligase that negatively regulates PHT1. To further optimize Pi acquisition, the PHT pathway promotes the development of lateral roots and root hairs, effectively increasing the root surface area and enhancing the plant’s ability to forage for Pi (Fang et al., 2024). While the specific targeting signals for PHT1 transporters remain an active area of research, it is known that their correct localization to the plasma membrane is critical for their function in Pi uptake. The N-terminal region of PHT1 transporters contains important determinants for their trafficking and plasma membrane targeting (Bayle et al., 2011). Although the precise motifs or sequences involved may vary among PHT1 transporter isoforms and plant species, it is understood that these targeting signals facilitate their appropriate placement in the plasma membrane. Recent research has also revealed the involvement of intracellular vesicle trafficking in the membrane localization of PHT1 transporters. It has been proposed that the transporters are initially synthesized in ER and subsequently targeted to the Golgi apparatus, where they undergo post-translational modifications (Victor Roch et al., 2019). These transporters exhibit a high affinity for Pi, enabling them to scavenge even trace amounts of Pi in the soil solution. For example, in millets, the expression of *PHT1;2* and *PHT1;3* genes significantly increase under Low-P conditions. This elevation in transporter activity plays a vital role in PUE and ultimately contributes to improved crop yields in nutrient-poor soils (Maharajan et al., 2019). Despite the recognized importance of various PHT1 family members, there is still a significant gap in understanding their specific functions and regulatory mechanisms. For example, studies on ryegrass have shown that *LpPHT1;4* functions as a high-affinity transporter activated by P starvation, while *LpPHT1;1* is a low-affinity transporter with limited sensitivity to P availability (Parra-Almuna et al., 2020). This distinction raises important questions about leveraging high-affinity PHT1 transporters in breeding programs to enhance P uptake, as well as the strategies needed to improve the responsiveness of low-affinity transporters in P-deficient environments. These findings highlight an urgent need to manipulate these transporter genes in crop species, particularly in low-input farming systems facing P scarcity. Research on maize has identified *ZmPHT1;1* as a key candidate gene for improving PUE through genome-wide association studies (GWAS) (Li et al., 2021). This not only points to the potential for targeted breeding strategies but also emphasizes the need for extensive genomic research across various crops.

It is evident that PHF1 is involved in intracellular trafficking of multiple PHT1 family proteins, for example, Mutations in the *PHF1* locus result in a severe decrease in Pi influx, as the mutant allele leads to an abnormal accumulation of PHT1;1, PHT1;2 and PHT1;4 in the ER (**Figure 1**). Similar targeting defects have been observed in rice, where a mutant of *OsPHF1* affects several members of the PHT1 family. These include the low affinity Pi transporter OsPT2 and the high affinity Pi transporter OsPT8 (Chen et al., 2015). Recent studies have revealed the significant role of *OsPHT1;3* in Pi absorption, root-to-shoot translocation, and remobilization within the plant, particularly under extremely low Pi conditions (Chang et al., 2019). Moreover, it was discovered that *OsPHT1;3* physically interacts with *OsPHT1;2*, suggesting a potential cooperative mechanism in Pi transport. Similar findings have been documented for Arabidopsis PHT1;1 and PHT1;4 proteins, highlighting their ability to form both homomeric and heteromeric complexes (Fontenot et al., 2015). Although the precise biological significance of these interactions remains largely unknown, these findings strongly indicate the possibility of PHT1 proteins forming oligomeric structures in both monocots and dicots. Moreover, recent studies in rice, identify that WRKY21 and WRKY108 have been found to activate the expression of *OsPHT1;1* under Pi-sufficient conditions to promote Pi accumulation (Zhang et al., 2021a).

The PHT1 pathway is well-documented for its essential role in high-affinity Pi uptake, other PHT transporter families such as PHT2, PHT3, PHT4, and PHT5 also play significant roles in maintaining Pi homeostasis in plants (Victor Roch et al., 2019). The PHT2 family has been implicated in the regulation of Pi levels under varying environmental conditions. In poplar (*Populus trichocarpa*), *PtPHT2;1* predominantly expresses in roots, cambium-phloem, mature petioles, and dormant buds, while *PtPHT2;2* is mainly expressed in roots and leaves under Low-P conditions (Zhang et al., 2016). These expression patterns indicate their potential as markers for high PUE genotypes. In wheat, knockdown of the *TaPHT2;1* transporter caused a significant reduction in Pi accumulation, regardless of Pi availability (Guo et al., 2013). Notably, *TaPHT2;1* enhances Pi concentration in chloroplasts, facilitating Pi transfer from the cytosol (Guo et al., 2013, Victor Roch, Maharajan et al., 2019). Similarly, overexpressing *OsPHT2;1* in rice led to increased biomass and elevated leaf Pi levels (Shi ShuLin et al., 2013). However, characterization of the PHT2 family in plants beyond poplar, including Arabidopsis, remains limited. while PHT3 transporters are known to facilitate Pi transport across membranes in different tissues, particularly in the mitochondria, where PHT3;1 plays a critical role in maintaining Pi homeostasis essential for ATP synthesis and redox balance (Jia et al., 2015). The widespread expression of PHT3;1 across various tissues underscores its significance in supporting mitochondrial functions, especially in root cells, whereas the more restricted expression patterns of PHT3;2 and PHT3;3 in green and reproductive tissues indicate a specialized role in plant development (Jia et al., 2015). The PHT4 and PHT5 transporter families exhibit distinct physiological roles that are crucial for enhancing PUE in plants and adapting to Low-Pi conditions. PHT4 transporters, such as PHT4;1, are primarily involved in the remobilization of Pi during plant development. They facilitate Pi transport in thylakoid membranes, which is essential for ATP synthesis, helping maintain adequate internal Pi levels during periods of high demand, particularly during photosynthesis (Wang et al., 2014a; Karlsson et al., 2015). Conversely, the PHT5 family, localized mainly in vacuoles, plays a critical role in accumulating and regulating intracellular Pi levels. Under Low-P conditions, these transporters, like PHT5;1, prevent Pi depletion by facilitating the uptake from vacuolar stores (Liu et al., 2016; Victor Roch et al., 2019). Disrupting PHT5;1 in plants leads to a noticeable decrease in total Pi content (Liu et al., 2015). By functioning in concert, these transporter families enable plants to optimize PUE, ultimately improving resilience to Low-P conditions and paving the way for the development of P-efficient crops that can reduce dependence on Pi fertilizers (**Table 1**).

#### 3.1.3 MYB62 and WRKY75 pathways: transcription factors involved in Pi homeostasis and stress response

*MYB62* is a member of the MYB transcription factor family, specifically belonging to the MYB-R2R3 subfamily. It is predominantly localized in the nucleus, indicating its role in transcriptional regulation (Abdullah et al., 2023). Specifically, *MYB62* controls the expression of PTs like *PHT1;1*, *PHT1;4*, and *PHT1;8* in plant roots. Furthermore, *MYB62* modulates the expression of genes involved in intracellular P transport and redistribution, enabling appropriate allocation of P in various plant tissues (**Figure 1**). Notably, *MYB62* not only activates genes associated with P acquisition and mobilization but also influences the expression of phosphatases, including PAPs and ribonucleases (Abdullah et al., 2023). These phosphatases release P from organic compounds, promoting its recycling for plant utilization. Additionally, *MYB62* regulates P homeostasis through its modulation of microRNAs, specifically affecting the expression of miR399 and miR827, which target specific transcripts such as *PHO2* and *IPS1* (**Figure 1**) (Yang and Finnegan, 2010). The *MYB62* pathway plays a critical role in regulating Pi homeostasis and stress responses across various crops, although its mechanisms and interactions vary among species. In Arabidopsis overexpression of *MYB62* in plants leads to a gibberellin-deficient (GA-deficient) phenotype, as transcript levels of GA biosynthetic genes and PSI genes decrease in *MYB62*-overexpressing plants (Devaiah et al., 2009). Furthermore, *MYB62* is induced in P-limited leaves and suppresses the expression of shoot PSI genes, including *SOC1* (**Figure 1**) which encodes a crucial molecular regulator of flowering time in plants. *SOC1* acts as a central integrator of flowering signals by promoting floral transition in response to environmental cues, thus linking nutrient status to reproductive development. This suppression of *SCO1* by *MYB62* highlights its critical role in integrating Pi-starvation responses with GA-signaling, potentially influencing not only root and shoot development but also the timing of flowering under nutrient-limiting conditions. Consequently, the interaction between *MYB62* and *SCO1* presents a fascinating avenue for understanding how plants adapt their growth and reproductive strategies in response to fluctuating nutrient availability (Borges Osorio, 2018).

Unlike *AtPHR1*, *AtMYB62* expression is specifically induced by Pi starvation in the leaves of young seedlings, unaffected by deficiencies in potassium, iron, or nitrogen (Yang and Finnegan, 2010; Borges Osorio, 2018). The Rapid loss of *AtMYB62* transcripts upon Pi resupply, indicating its significant role in regulating Pi deficiency-related genes involved in Pi signaling, high-affinity Pi transport, and mobilization. Additionally, Overexpression of *AtMYB62* under Pi-sufficient conditions induces responses similar to Pi starvation, including increased anthocyanin production, reduced primary root length, and increased root acid phosphatase activity (Yang and Finnegan, 2010). Exogenous GA partially rescues the phenotype, suggesting that *MYB62* may regulate Pi starvation responses through changes in GA concentration. In rice, for instance, *MYB62*, particularly *OsMYB2P-1*, modulates Pi responses by regulating gene expression and root architecture under low Pi conditions (Dai et al., 2012). Similarly, in wheat, *MYB62* contributes to abiotic stress tolerance (e.g., drought and salinity) and Pi uptake by regulating stress-responsive genes (e.g., *TaPHT1;2 and TaPHT1;4*) and other Pi-related genes such as *TaIPS1* and *TaSPX1*. These roles are reflected in the phenotypic differences between P-efficient and P-inefficient wheat genotypes, with the former exhibiting greater root biomass and length (Zheng et al., 2020). Recent studies in maize further highlight *MYB62* as a key regulator of Pi homeostasis, interacting with genes linked to root architecture, including *Zm00001d002837* and *Zm00001d002842*, which may promote or suppress their expression under Pi-deficient conditions. Additionally, *MYB62* interacts with transcription factors like ARF4, ARF7, ARF10, and bZIP11, which are crucial for root development and Pi uptake (Rajput et al., 2024), thereby extending its regulatory network to legumes, where *MYB62* may also influence nodulation signaling pathways, impacting both nitrogen and Pi acquisition (Mishra et al., 2024).

In addition to *MYB62*, several other MYB transcription factors are integral to P uptake and PUE in plants (**Table 2**). For example, *AtMYB2* functions as a transcriptional activator of miR399, significantly influencing Pi starvation signaling in Arabidopsis (Baek et al., 2013a), which in turn affects root architecture under P-deficient conditions. Similarly, *MdMYB2* regulates P assimilation while also impacting plant growth and flowering in *Malus*, thereby establishing a critical connection between P availability and developmental processes (Peng et al., 2020). Moreover, *TaMYB4-7D* enhances the expression of P transporter genes in wheat, thereby markedly improving P efficiency under low P conditions (Luo et al., 2024). Additionally, *GmMyb73* acts as a negative regulator of Pi-deficiency tolerance in soybean (Hu et al., 2024), underscoring the diverse regulatory mechanisms by which MYB transcription factors mediate responses to P availability across various plant species (**Table 2**). On the flip side, the WRKY75 pathway is involved in stress response, including responses to biotic and abiotic stresses (**Table 2**, **Figure 1**) (Kurt and Filiz, 2020; Huang et al., 2022; Gao et al., 2023). Recently, Kurt et al. conducted a gene co-expression network (GCN) analysis and found that *Glyma.19G094100* and *Glyma.16G054400* seed genes, orthologs to Arabidopsis *WRKY75*, have a direct connection to P deficiency, underscoring the significance of this pathway in nutrient stress responses (Kurt and Filiz, 2020). A root hair-specific *WRKY75* gene has been discovered to have significant effects on the transcriptional cross-talk among Pi starvation, phytohormones, and biotic stress signaling pathways (Baek et al., 2017). *WRKY75* mutation suppresses the transcription of PSI genes, including phosphatases, *Mt4/TPS1*-like genes, and Pi transporters (**Figure 1**) (Devaiah et al., 2007).

**Table 2:** Functional characterization of MYB and WRKY genes.

Gene Family	Member Genes	Function	Functional Role and Characterization	Method of Characterization	Plant Species	Reference
MYB Family	*MYB62*	Transcription Factor	Involved in the regulation of PHR; negatively regulates PHR1 and influences root architecture through interactions with ARFs and bZIP11.	DNA-protein interaction analysis, RNA-seq data mining, STRING analysis for protein-protein interactions.	Maize	(Rajput et al., 2024)
	*MYB62*	Transcription Factors	Upregulated during Pi-starvation; regulates root architecture, Pi uptake, and gibberellin biosynthesis.	Expression analysis, promoter binding assays, transgenic lines.	Arabidopsis	(Baek et al., 2013b)
	*MdMYB2 (MDP0000823458)*	Pi starvation response	Regulates PSI gene expression; connects Pi starvation and GA signaling; overexpression alters root architecture and anthocyanin accumulation.	Cloning, ectopic expression analysis in Arabidopsis, gibberellin application.	Apple	(Yang et al., 2020)
	*AtMYB62*	R2R3-type transcription factor	Negative regulator of GA biosynthesis; specifically induced in leaves during Pi deficiency; impacts root architecture and GA-related processes.	RT-qPCR, overexpression and mutant analysis, phylogenetic analysis, GUS staining.	Arabidopsis	(Devaiah et al., 2009)
	*McMYB10*	Transcription factor	Regulates anthocyanin biosynthesis genes in response to Pi deficiency; expression is modulated by epigenetic factors such as *McHDA6 and McMET1.*	RT-qPCR, promoter methylation analysis, histone activity assessment	Crabapple	(Peng et al., 2020)
	*MYB1*	R2R3-type transcription factor	Induced by Pi starvation; regulates Pi uptake and root development; negatively affects Pi transporter expression.	RT-qPCR, subcellular localization (GFP) analysis, transgenic and mutant analysis	Rice	(Gu et al., 2017)
	*AtMYB2*	Transcription factor	Acts as a transcriptional activator for miR399; influences Pi signaling; induces root architectural changes.	Promoter binding assays, comparative analysis of transgenic and wild-type plants	Arabidopsis	(Baek et al., 2013a)
	*StMYB44*	Negative regulator of Pi-transport	Downregulated by Pi-starvation; interacts with transcription factors to suppress *StPHO1* expression.	RNA-seq, qRT-PCR validation, protein-protein interaction assays	Potato	(Zhou et al., 2017)
	*AtMYB44*	Negative regulator of Pi-transport	Functions as a mobile mRNA responding to Pi starvation; negatively regulates PHT1;2 and PHT1;4 expression in roots.	qRT-PCR for expression validation.	Arabidopsis	(Olukayode et al., 2023)
	*TaMYB4-7D*	Transcription Factor	Enhances expression of Pi-transporter genes, improving P efficiency under Low-P conditions.	Transcriptome analysis, differential gene expression analysis	Wheat	(Luo et al., 2024)
	*GmMyb73*	Negative regulator of Pi-deficiency tolerance	Interacts with *GmGDPD2* promoter, inhibiting its expression and regulating low-P tolerance in soybean.	Yeast one-hybridization, luciferase complementation assays	Soybean	(Hu et al., 2024)
	*TaPHR3-A1* (a member of the MYB family)	Regulator of Pi-signaling	Positively regulates Pi uptake; enhances growth and yield-related traits under low Pi conditions in various plants.	Ectopic expression in Arabidopsis/rice, phenotypic assessment, linkage analysis	Wheat	(Zheng et al., 2020)
	*SlPHL1* (a MYB-CC transcription factor)	Regulator of PSR	Enhances Pi-starvation responses through various mechanisms; compensates for *AtPHR1* mutation effects.	Overexpression studies in Arabidopsis and tomato, EMSA, interaction analysis.	Tomato	(Campo and San Segundo, 2020)
	*RLI1/HINGE1* (a MYB transcription factor)	Modulator of nitrate-induced phosphate response (NIPR)	Functions downstream of PHR2; enhances PSI gene transcription; competes with SPX proteins for binding to PHR2.	RNA-seq, dual-luciferase assays, co-immunoprecipitation, transient assays.	Rice	(Zhang et al., 2021)
WRKY Family						
	*AtWRKY75*	Transcription factor involved in Pi homeostasis	Enhances Pi translocation by interacting with the *AtPHT1;5* promoter	Promoter analysis, binding affinity assessment	Arabidopsis	(Shee et al., 2022)
	*AtWRKY75*	Positive regulator of Pi acquisition and stress response	Improves root growth and Pi-uptake efficiency through overexpression.	Transgenic lines, gene expression analysis, physiological measurements	Arabidopsis	(Devaiah et al., 2007)
	*WRKY75*	Regulator of root architecture	Induced by P starvation; negatively impacts root architecture in response to P deficiency.	Expression analysis, microarray	Maize	(Lin et al., 2013)
	*WRKY75*	Transcription factor in stress response	Regulates gene expression under P-deficiency.	RNA-seq, confirmed via qPCR or functional assays	soybean	(Gao et al., 2023)
	*Glyma.19G094100 Glyma.16G054400* (orthologs to WRKY75),	Transcription factors in stress tolerance	Identified in co-expression network under Low-Pi; linked to P-deficiency.	Co-expression network analysis, promoter analysis, methylation profile analysis	soybean	(Kurt and Filiz, 2020)
	*OsWRKY74*	Modulator of Pi starvation tolerance	Overexpression enhances root and shoot biomass; higher P concentration in roots and shoots.	Overexpression & RNAi lines, quantitative RT-PCR, hydroponic & soil pot experiments	Rice	(Dai et al., 2016)
	*AtWRKY42*	Regulator of Pi homeostasis	Controls *PHO1* and *PHT1;1* gene expression in Arabidopsis.	Expression analysis, mutant line characterization.	Arabidopsis	(Su et al., 2015)
	*LusWRKY7, LusWRKY2, LusWRKY48*	Regulators of P deficiency response	Upregulated under low-P, enhancing root architecture and stress resilience via hormonal modulation.	qRT-PCR	Flax	(Huang et al., 2022)
	*GmWRKY46*	Enhancer of Pi starvation tolerance	Promotes root development; primarily localized in the nucleus with transcriptional activation.	RNA-seq, RT-qPCR, Y1H, ChIP-qPCR, transgenic plant analysis	Soybean	(Li et al., 2021a)
	*TaWRKY74* (three alleles: *TaWRKY74-A, TaWRKY74-B, TaWRKY74-D*)	Regulator of tiller number	Influences axillary bud development under low-P stress.	RNA-seq, RNA interference (RNAi), gene expression assays	Wheat	(Li et al., 2024)

There are 72 WRKY gene family members in *Arabidopsis thaliana*, 4 members are determined to participate in responding to low P, containing *AtWRKY75* (Devaiah et al., 2007), *AtWRKY6* (Chen et al., 2009), *AtWRKY45* (Wang et al., 2014) and *AtWRKY42* (Su et al., 2015). Research has shown that *AtWRKY75* (Devaiah et al., 2007) and *AtWRKY45* (Wang et al., 2014) are positive TFs for P deficiency response, but *AtWRKY42* (Su et al., 2015) and *AtWRKY6* (Chen et al., 2009) are negative regulators of P deficiency response in Arabidopsis (Huang et al., 2022). Interestingly, *WRKY75* and *WRKY45* exhibit a mutual negative regulatory relationship in terms of auto-regulation. *WRKY75* binds to two W box elements within the *WRKY45* promoter, repressing the transcription of the *WRKY45* gene (Wang et al., 2014). This finding point out the intricate nature of the interplay between WRKY transcription factors in coordinating various stress responses. Recently Gao et al. (2023) have identified the crucial roles of *WRKY75* and *MYB86*, members of the MYB transcription factor family, in the response of wild soybean to P deficiency (Gao et al., 2023). The upregulation of *WRKY75* and downregulation of *MYB86* contribute to the enhanced resistance of wild soybean against low P stress (**Figure 1**). This regulation positively affects the expression of high affinity PTs and phosphatase genes, facilitated by *WRKY75* (Kurt and Filiz, 2020). Additionally, it leads to increased anthocyanin synthesis, which plays a protective role by absorbing ultraviolet light and safeguarding chloroplast membranes from damage, thus aiding the resistance against low P stress. Furthermore, the negative regulation of *MYB86* by these transcription factors helps reduce stress-induced damage (Gao et al., 2023). Beyond *WRKY75*, other members of the WRKY transcription factor family also play critical roles in mediating plant responses to P deficiency, highlighting the broad significance of this gene family in nutrient stress management (**Table 2**). For instance, recent transcriptome analyses in wheat have identified *TaWRKY74* as a key factor influencing tiller development under low P stress, as it regulates hormonal pathways, particularly ABA and auxin signaling, essential for optimizing plant architecture and nutrient uptake (Li et al., 2024). Similarly, in flax, the expression of *LusWRKY* genes (*LusWRKY7, LusWRKY22, LusWRKY48*, and *LusWRKY71*) was significantly upregulated in response to P deficiency, suggesting that these transcription factors facilitate root adaptations to enhance P acquisition by promoting lateral root growth and altering hormone levels (Huang et al., 2022). Furthermore, studies on rice demonstrate that *OsWRKY74* enhances tolerance to Pi starvation by modulating root system architecture and activating P-responsive genes, also indicating potential crosstalk with iron and nitrogen deficiency responses (Devaiah et al., 2009). The *MYB62* and *WRKY75* transcription factors represent vital components in the plant regulatory network governing Pi homeostasis and stress response. Their intricate interactions and roles suggest potential targets for enhancing P efficiency and stress tolerance in crops (**Table 2**).

#### 3.1.4 SPX pathway: negatively regulating phosphate transport and signal transduction

The SPX pathway is essential for regulating P transport in plants. It involves the SPX domain-containing protein family, which acts as a negative regulator of Pi signaling. The SPX domain binds to small molecules, such as inositol polyphosphate signaling molecules (InsPs) and interacts with other proteins such as PHR1 to control Pi signaling in plants (**Figure 1**), especially in Pi-deficient cells (Jung et al., 2018).

SPX genes, such as *AtSPX1* in Arabidopsis and *OsSPX1* in rice, play significant roles in regulating PSI genes (**Figure 1**). In Arabidopsis, *AtSPX1* acts as a P-dependent suppressor of *AtPHR1* (Zhou et al., 2015), while in rice, *OsSPX1* inhibits P uptake and P-starvation signaling through negative feedback regulation (Wang et al., 2009). The interaction between *OsSPX1* and *OsPHR2* also influences P concentration and PSI gene expression (Lv et al., 2014).

Legume plants, such as soya beans, also utilize SPX genes to regulate P acquisition and transport, with their expression being highly sensitive to low P conditions, for example *GmSPX-RING1* affects P efficiency by negatively regulating P concentration in soybean hairy roots (Du et al., 2020). Wheat possesses *TaSPX3*, which is strongly induced during low P stress and downregulated upon P supply restoration. Additionally, the SPX subfamily in maize has also been found to play pivotal roles in P stress sensing and response. The SPX subfamily, especially *ZmSPX4.1* and *ZmSPX4.2*, were significantly induced under P-deficient conditions and exhibited remarkably different expression patterns in low Pi sensitive and insensitive cultivars of maize (Kant et al., 2011).

A recent study revealed the interaction of *OsSPX4* with *OsbHLH6* and their participation in the phosphate-starvation response (PHR) (He et al., 2021) in rice. This MYB-like transcription factor is homologous to phosphorus starvation response 1 (PSR1), which participates in the P sensing process in *Chlamydomonas reinhardtii* (Bari et al., 2006). *PHR1* regulates the expression of SPX genes, by binding to their promoters through the cis-element PHR1-binding sequence (P1BS; GNATATNC) (**Figure 1**) (Rubio et al., 2001; Bari et al., 2006). The mechanisms governing SPX protein function are not limited to modulation of P transport; they also extend to the intricate regulatory networks involving nitrogen status through nitrate-inducible (NIGT1) proteins. These NIGT1 proteins directly bind to the promoters of several SPX genes, including *SPX1* and *SPX4*, repressing their expression and thereby integrating nitrogen signaling with Pi starvation signaling to optimize nutrient uptake PSR (Ueda et al., 2020).

Overall, the SPX pathway’s multifaceted regulatory roles affirm its significance in plant responses to varying nutrient levels, emphasizing its potential as a target for enhancing PUE in agricultural systems.

### 3.2 The intricate regulation of phytohormones in influencing PUE and allocation

The general responses of plants to P deficiency include a multifaceted set of strategies such as morphological, physiological changes and molecular regulation of gene expression. It has been well documented that numerous phytohormones and signalling molecules are involved in the responses to P deficiency in plants, including auxin (Wang et al., 2014b), ethylene (Zhang et al., 2020; Marro et al., 2022), nitric oxide (NO) (Zhu et al., 2016a; Zhu et al., 2017), strigolactones (Santoro et al., 2022) and abscisic acid (ABA) (Fang Zhu et al., 2018; Castro-Valdecantos et al., 2023).

Auxin plays a crucial role in regulating plant responses to P deprivation. Specifically, research has shown that auxin signaling is integral in modifying root architecture under low P conditions, particularly by promoting the growth of root hairs (Li et al., 2019). Maintaining auxin homeostasis is essential for elongating root hairs in P-deficient conditions. However, the molecular mechanisms governing root hair elongation under P stress are not well understood. The concentration of auxin in plant tissues can be controlled through both biosynthesis (TAA1) and transport (AUX1) mechanisms (**Figure 2**), allowing plants to adapt to different P availability by regulating their responses to nutrient stress (Bhosale et al., 2018). Additionally, a connection between auxin signaling and the response to P starvation in various plant species has been discovered. For example, in Arabidopsis, auxin has been linked to the response to P starvation, with mutants displaying reduced auxin sensitivity and altered P uptake (Huang et al., 2018). Similarly, in rice, AUXIN RESPONSE FACTOR 16 (*ARF16*) acts as an integrator of both auxin and P starvation signals to regulate root development and nutrient uptake. Loss of function of *OsARF16* leads to decreased root growth, reduced sensitivity to auxin, and Low-P conditions (Shen et al., 2013). Additionally, *ARF16* is associated with the cytokinin signaling pathway, which modulates P uptake and utilization. Cytokinin may repress the response to P starvation by increasing intracellular Pi content (Shen et al., 2014). Conversely, the mutant lacking *OsARF12* shows increased P concentrations and symptoms of P toxicity, suggesting a loss of regulation over P absorption and translocation. *ARF12* negatively regulates P uptake and translocation through the *PHT1* gene family. The transcription of *PHT1* genes is regulated by auxin through the presence of auxin-related cis-acting elements in their promoters (Baek et al., 2017). Auxin receptor TRANSPORT INHIBITOR RESPONSE1 (*TIR1*) and AUXIN RESPONSE FACTOR19 (*ARF19*) are also involved in enhancing auxin sensitivity in plants under Low-P conditions. *ARF19* promotes root hair elongation by regulating the expression of genes like ROOT HAIR DEFECTIVE 6-LIKE 2 (*RSL2)* and ROOT HAIR DEFECTIVE 6-LIKE 4 (*RSL4)*, which encode transcription factors that promote root hair initiation and elongation (Yi et al., 2010). Bhosale et al. (2018) demonstrate that Low-P levels increase the levels of auxin (IAA) in the root apex, facilitated by TAA1-mediated auxin synthesis and AUX1-dependent auxin transport. This increase in IAA levels induces the expression of *ARF19* in the root apex, resulting in the induction of *RSL2* and *RSL4* in the elongation and differentiation zones, respectively, promoting root hair elongation (Bates and Lynch, 1996).

P deficiency also decreases GA and activates the DELLA-mediated signaling pathway, which impacts root hair and lateral root growth (Jia et al., 2017). Similarly, the SUMO E3 ligase SIZ1 is associated with P starvation responses and may negatively regulate auxin patterning, thus affecting root architecture under Low-P conditions. On the other hand, the cotton defense-related gene *GbWRKY1* has recently been identified as a positive regulator of the P response by enhancing auxin sensitivity and driving modifications in the root system (Miura et al., 2011). Importantly, *GbWRKY1* appears to function independently of both *SIZ1* and *PHR1* in response to P (Wang et al., 2014b). The involvement of auxin signaling through *ARF12*, *ARF16*, and *ARF19* in PUE underscores the importance of understanding the mechanisms underlying nutrient uptake and utilization.

Numerous studies have shown that abscisic acid (ABA) plays a crucial role in regulating genes involved in P uptake and utilization under low-P conditions (Nagatoshi et al., 2023). ABA accumulation, perceived by PYRABACTIN RESISTANCE1/PYR1-LIKE/REGULATORY COMPONENTS OF ABA RECEPTOR (PYR/PYL/RCAR) receptors, can upregulate the expression of ABA biosynthesis genes (*ABA1*, *NCED3*, *NCED6*, *ABA2* and *AAO3*) and ABA-glucosyl ester deconjugation genes (*BG1* and *BG2*), while repressing ABA catabolism genes (*CYP707A1* and *CYP707A3*) (**Figure 2**) (Zhang et al., 2022). The transcription factor *ABI5* (basic leucine zipper (bZIP) transcription factor), a central player in the ABA signaling pathway, facilitates P acquisition by activating the expression of *PHT1;1* and *PHT1;4* (Carles et al., 2002). ABA also mediates stomatal closure, which contributes to the overall regulation of P utilization in plants (Castro-Valdecantos et al., 2023). P-starved plants show an increase in foliar ABA concentration, which regulates stomatal closure. Other hormones like Jasmonic acid (JA) and *trans*-zeatin (TZ) may also affect stomatal opening and ABA sensitivity in P-starved plants (**Figure 2**). JA has been suggested to interact synergistically with ABA to cause stomatal closure (de Ollas and Dodd, 2016). ABA accumulation in leaves is believed to be responsible for P-starvation-induced stomatal closure. ABA also influences P remobilization of root cell wall in rice independent of nitric oxide and ethylene (Fang Zhu et al., 2018). It inhibits the re-utilization of P stored in root cell walls and decreases the expression of a high-affinity Pi transporter, resulting in reduced shoot soluble P content (Fang Zhu et al., 2018). These interactions between ABA and other hormones highlight the complexity of regulating P uptake and utilization.

However, the exact mechanisms by which ABA regulates gene expression under low P conditions are not fully understood. Further studies are needed to elucidate the hormonal crosstalk involved in these responses and to explore other signaling pathways and transcription factors that may be involved, such as the mitogen-activated protein kinase cascade and ABA-responsive element binding transcription factors.

Ethylene also plays a crucial role in helping plants adapt to P starvation by regulating changes in the root system’s structure (Dubois et al., 2018). Under P-deficient conditions, plants produce more ethylene, which acts as a regulator for root system architecture (RSA) (Liu, 2021). It inhibits the extension of the principal root and stimulates the development and elongation of lateral roots. The production of ethylene is highest in adventitious roots and is inhibited by Low-P stress, redirecting carbon allocation to adventitious roots at the expense of other roots. Pectin, the major component of plant cell walls, is known to respond to P starvation, and its concentration determines a plant’s resistance to Low-P stress. Ethylene increase pectin concentrations and pectin methylesterase (PME) activity in the root cell wall under P-deficient conditions, enhancing PUE. For instance, groundnut’s root cell walls contain “contact reaction” pectin (**Figure 2**), allowing it to efficiently acquire soil P in P-deficient soil (Zhu et al., 2016b). Additionally, ethylene also enhances pectin concentrations in the root cell walls of rice under P-deficient conditions. SAMS1,(an enzyme involved in ethylene biosynthesis) increase pectin concentrations and PME activity in tomato under P-deficient conditions (**Figure 2**), suggesting that optimizing the interactions between ethylene and other metabolic pathways may enhance plant response to P stress (Wang et al., 2022).

Ethylene also promotes root hair development by influencing auxin biosynthesis and transport, regulating cell wall modifications (Stepanova et al., 2011; Song et al., 2016), and altering JA biosynthesis. Overexpression of *GmETO1*, a member of the *ETO1* family strongly induced by Pi deficiency, significantly enhances Pi deficiency tolerance. This enhancement is achieved by increasing the proliferation and elongation of hairy roots, as well as improving Pi uptake and use-efficiency. Conversely, silencing of *GmETO1* leads to opposite results (Zhang et al., 2020). Ethylene improves P acquisition by mobilizing phosphate (Po), activating acid phosphatase (APase) activity, promoting P remobilization, and inducing the transcription of PTs (**Figure 2**) (Zhu et al., 2016b). Fine-tuning ethylene biosynthesis or signaling is a practical approach to enhance plant P utilization without compromising desirable agronomic traits. However, directly manipulating the enzymes responsible for regulating ethylene biosynthesis (ACS and ACO) can have negative effects on traits such as plant height, fruit ripening, and senescence (Ye et al., 2018).

Strigolactones (SLs) are carotenoid-derived phytohormones primarily synthesized in plant roots in response to nitrogen and/or P deficiencies in various plant species (Marro et al., 2022). They function as signaling molecules that elicit various morphological, physiological, and biochemical responses, enabling plants to adapt to Low-P conditions (**Figure 2**).

Recent studies have demonstrated increased SL biosynthesis and exudation in many species under P-starved conditions. For instance, Pi-deficiency affects leaf angle and root SL production in rice. The up-regulation of SL-signaling genes, such as *D3* and *D14*, under P deficiency (**Figure 2**) suggests that endogenous SLs may mediate the plant’s leaf angle sensitivity to low Pi levels (Ruan et al., 2018). Moreover, SLs significantly influence the expression of key P signaling and regulatory genes, including high-affinity P transporters and phosphorus-hydrolyzing enzymes in tomato plants (Santoro et al., 2021). Marro et al. (2021) Proposed mechanisms which suggest that SLs act on the SPX-PHR1 complex, which releases the transcriptional activator PHR1. Furthermore, Santoro et al. (2021) proved that, this interaction leads to an increase in mature miR399 levels and subsequent expression of *TPSI1*, a gene involved in miR399 sequestration. This process reduces the transcript levels of the PSR suppressor PHO2, activating the phosphate starvation response (PSR) pathway and inducing the expression of genes encoding PTs for enhanced P acquisition (Gamir et al., 2020; Santoro et al., 2021; Marro et al., 2022). Furthermore, SLs also exert regulatory control over other P starvation-related hormones, such as auxin, ethylene, and BR, through complex interplay (Gamir et al., 2020). Under Low-P conditions, acclimation involves the repression of ethylene biosynthesis by ACC and ACC oxidase, and the activation of auxin signaling. Additionally, SLs regulate the expression of genes involved in GA production, which is connected to BR through the positive regulator BES1 (Wang et al., 2021b). These interactions highlight the intricate network of hormone cross-talk underlying plant responses to P deficiency (**Figure 2**).

GA does not directly regulate P uptake from the soil to the plant. However, it plays a critical role in the development of arbuscular mycorrhizal (AM) associations in plants, which in turn enhances P uptake. GA can either inhibit or promote AM colonization and can positively interact with symbiotic responses in the host root. The balance between ABA and GA is crucial for AM formation, as a high ABA/GA ratio can reduce arbuscule abundance (**Figure 2**). Interestingly, certain bacteria can produce GAs, which enhance GA production in plants, further promoting AM symbiosis. On the other hand, blocking GA biosynthesis can decrease AM fungal colonization and hyphal branching in the host root, emphasizing the importance of GA in arbuscular mycorrhiza development. The intricate interactions between hormones and microorganisms in the rhizosphere warrant further research for a thorough understanding in response to P deficiency. These interactions enhance the understanding of plant physiology and have significant implications for agricultural practices aimed at improving PUE in crops.

### 3.3 Soil enzymes and phosphorus uptake in low-P availability environments

Soil enzymes are essential for the uptake and utilization of P by plants, especially in conditions of Low-P availability. There are numerous enzymes found in soil, including phosphatase, phytase, nuclease, amylase, and cellulase. Among them phosphatase and phytase, are important for the uptake and utilization of P by plants, particularly in conditions of low P availability. Phosphatase enzymes help convert organic forms of P into inorganic forms that plants can absorb, while phytase enzymes help break down phytic acid, a form of organic phosphorus found in plant tissues and soil, making it available for plant uptake. The other enzymes mentioned, such as nuclease, amylase, and cellulase, may have other roles in soil processes but may not directly contribute to P uptake and utilization.

#### 3.3.1 Phosphatases enhancing PUE and inorganic phosphate fixation

Phosphatases are critical for the acquisition and utilization of Pi. Among various phosphatases, acid phosphatases (APase) play a pivotal role as they increase the bioavailability of Pi by breaking it down into a more accessible form (Zhang et al., 2010). The activity of APase is detected throughout development, and their release to the rhizosphere is a typical response of Phosphorus-deficient in higher plants (Lazali and Drevon, 2018). A study by Lu et al. (2016) identified *OsPAP10c*, a novel secreted APase in rice that belongs to a monocotyledon-specific subclass of Ia group PAPs and is specifically expressed in the epidermis/exodermis cell layers of roots (Lu et al., 2016). Overexpression of *OsPAP10c* resulted in a more than ten-fold increase in APase activity, which enhanced the plant’s efficiency in utilizing external organic P in turn.

Recently, it has been discovered that the expression of APase and phytase genes, as well as the activities of the corresponding enzymes, are positively correlated with the increases in both P use-efficiencies for N_2_ fixation and nodule O_2_ permeability in the rhizobial symbiosis in legumes (Lazali and Drevon, 2018). In soybean nodules, there is also evidence of the overexpression of numerous APase enzymes involved in Pi homeostasis, suggesting that the excretion of nodular APase may be stimulated by low P availability (Li et al., 2012). Similar findings have been identified in *Brassia napus* by (Zhang et al., 2010) and in various grasses such as *Axonopus afnis, Paspalum notatum*, and *Andropogon lateralis* (de Oliveira et al., 2018). These studies demonstrate the relationship between Apase activity and P remobilization in grasses with different growth rates. Particularly, higher growth rates are associated with elevated P uptake efficiency and increased remobilization of P due to higher demand, as observed in *A. afnis* (de Oliveira et al., 2018).

Another important subtype of APase found in plants is the Purple Acid Phosphatases (PAPs). These enzymes have a high affinity for organic phosphorus compounds, making them critical in optimizing plant growth and crop productivity, especially in soils with limited available P. The expression of PAPs and its homologues are primarily regulated at the transcriptional level by PHR1 (Sun et al., 2016; Mehra et al., 2017). Most of the PAPs have PHR1 binding sites in their promoters and are responsive to Pi deficiency (Bhadouria et al., 2017). Furthermore, plant growth regulators such as auxin, cytokinin, and ethylene have a role in regulating the expression of PAPs at the transcriptional level. For example, in rice mutants of the auxin-responsive transcription factor, *osarf12*, elevated the expression of 4 PAPs genes *OsPAP9b, OsPAP10a, OsPAP10c*, and *OsPAP27a* enhanced APase activity, suggesting that ARF12 influenced transcript abundance from PAP genes in P sufficient and deficient conditions (Wang et al., 2014b). Cytokinins negatively regulate the expression of PAPs, such as *AtPAP17* and *OsPAP10*, as shown in studies where exogenous cytokinins decreased the expression of these PAPs (Fang et al., 2009). Ethylene, a component of local cellular signaling in response to Pi levels in the rhizosphere, influences the regulation of *AtPAP10* expression in a Pi-dependent manner. Specifically, *AtPAP10* expression, protein accumulation, and activity increase under Pi-sufficient conditions in the presence of ACC (an ethylene precursor) (Zhang et al., 2014). Apart from their primary function in Pi homeostasis, PAPs are also involved in other processes such as root growth, symbiotic association, carbon metabolism, phospholipid hydrolysis, defense response, and cellular signaling. The intricate regulatory mechanisms and versatile roles of PAPs make them essential for optimizing plant growth and crop productivity, particularly in P-deficient soils. These phosphatases utilize a ping-pong mechanism to cleave the phosphodiester bond and coordinate with metal ions to increase bioavailability. The metal ions act as a catalyst and coordinate the substrate and the enzyme active site (Gottlin et al., 1998). The roles of various phosphatases in P-acquisition are essential, particularly in P-deficient environments. The capacity of soil enzymes to enhance P-uptake in these low-availability conditions indicates that optimizing enzyme activity is a vital strategy for enhancing PUE and overall crop productivity. A more profound understanding of these enzymes’ functionalities and efficiencies could bolster breeding programs focused on developing plant varieties that can efficiently utilize soil P. As research in this field advances, the prospects for innovative agricultural practices and enhanced crop varieties grow more promising.

#### 3.3.2 Phytate in facilitating ion-ligand complex formation to enhance PUE

Phytases are enzymes that facilitate the conversion of phytate to Pi, thereby releasing Pi and minerals in an accessible form, enhancing their absorption by plants (Sun et al., 2021). Phytate is a chemical derivative of inositol (myo-inositol hexabisphosphate) and is the most widely distributed form of P in the soil (Duong et al., 2018). For soil phytate to contribute to plant P nutrition, phosphate ester (C-O-P), phosphoanhydride (P-O-P), or phosphonate (C-P) must first be dephosphorylated through phytase-mediated hydrolysis. Desorption and solubilization are two approaches to improving phytate access (Richardson et al., 2011). Protons, organic acids, and phenolic acids can all desorbate or solubilize P in soil, with organic acids being the main solubilizer of the rarely available P (Richardson et al., 2011). Carboxylate groups found in organic acids play a multifaceted role in the mobilization of phytate. They facilitate this process by substituting Pi with a carboxylate anion, thereby promoting phytate solubility. Carboxylates further aid in phosphate anion desorption from the soil through ligand exchange mechanisms. Additionally, they contribute to the removal of P sorption sites by solubilizing iron (Fe) and aluminum (Al) through proton (H+) activity. Finally, carboxylates facilitate the dissolution of organic matter (OM) bound to P via Fe/Al-bridges, resulting in the release of Pi as part of the OM-Fe/Al-P complex (Gerke, 2010). Small quantities of Po added to the rhizosphere might act as a stimulator to phytic acid mineralization, thus improving plant P feeding. Both plant and microbial phytases play pivotal roles in the solubilization of phytate, which is a crucial step in P acquisition. In addition, transgenic plants, including maize, rice, and soybean, have been engineered to express phytase genes origin from microorganisms. This genetic modification aims to enhance plant P accumulation and promote increased biomass production, thus contributing to improved agricultural yields and sustainable crop growth (Puppala, 2018; Singh et al., 2020). It was found that the plants had significantly higher grain yields and P accumulation in their tissues, in transgenic maize expressing a fungal phytase gene (Xu et al., 2018). In the last decade, the genes involved in the synthesis of microbial phytases having a high affinity toward phytate have been utilized to produce transgenic plants. Phytase genes from bacteria, fungi, and yeasts such as *Bacillus subtilis, Selenomonas ruminantium, Escherichia coli, Aspergillus ficuum*, *Aspergillus niger*, and *Thermomyces lanuginosus* have been used to develop transgenic plants. The most studied *A. niger* enzyme has been successfully expressed in Arabidopsis, tobacco, wheat, maize, soybeans, alfalfa, and rapeseed (Valeeva et al., 2018). Several studies back up the advantages brought forth by transgenic types, such as the high expression of a PHY US417-related gene in Arabidopsis that led to increased growth and Po content without inducing inorganic phosphorus starvation-triggered (PSI) genes. Enhanced biomass and Pi were seen in plants co-cultured with ePHY overexpression when the PSI gene was suppressed (Belgaroui et al., 2016).

To fully understand how plants, acquire and utilize P, it is important to consider the various enzymes involved in this process. In addition to the well-studied acid phosphatases, purple acid phosphatases, and phytases, another enzyme called nucleases also play a role in releasing Po. Nucleases play a crucial role in the acquisition and utilization of P by plants. They break down nucleic acids, such as DNA and RNA, which are present in plant residues and soil organic matter. As they degrade nucleic acids, nucleases can hydrolyze phosphomonoesters and phosphodiester bonds, releasing Po that can be absorbed by plants (Solhtalab et al., 2022). This characteristic sets nuclease apart from other enzymes involved in P nutrition because they can release P sources other than soluble Po. Although the full significance of nucleases in plant P nutrition is still not clear, they may be more active in certain soil conditions, such as acidic soils with Low-P availability.

## 4 Interplay between rhizosphere microorganisms and factors influencing phosphorus efficiency in plant roots

**Figure 3:**
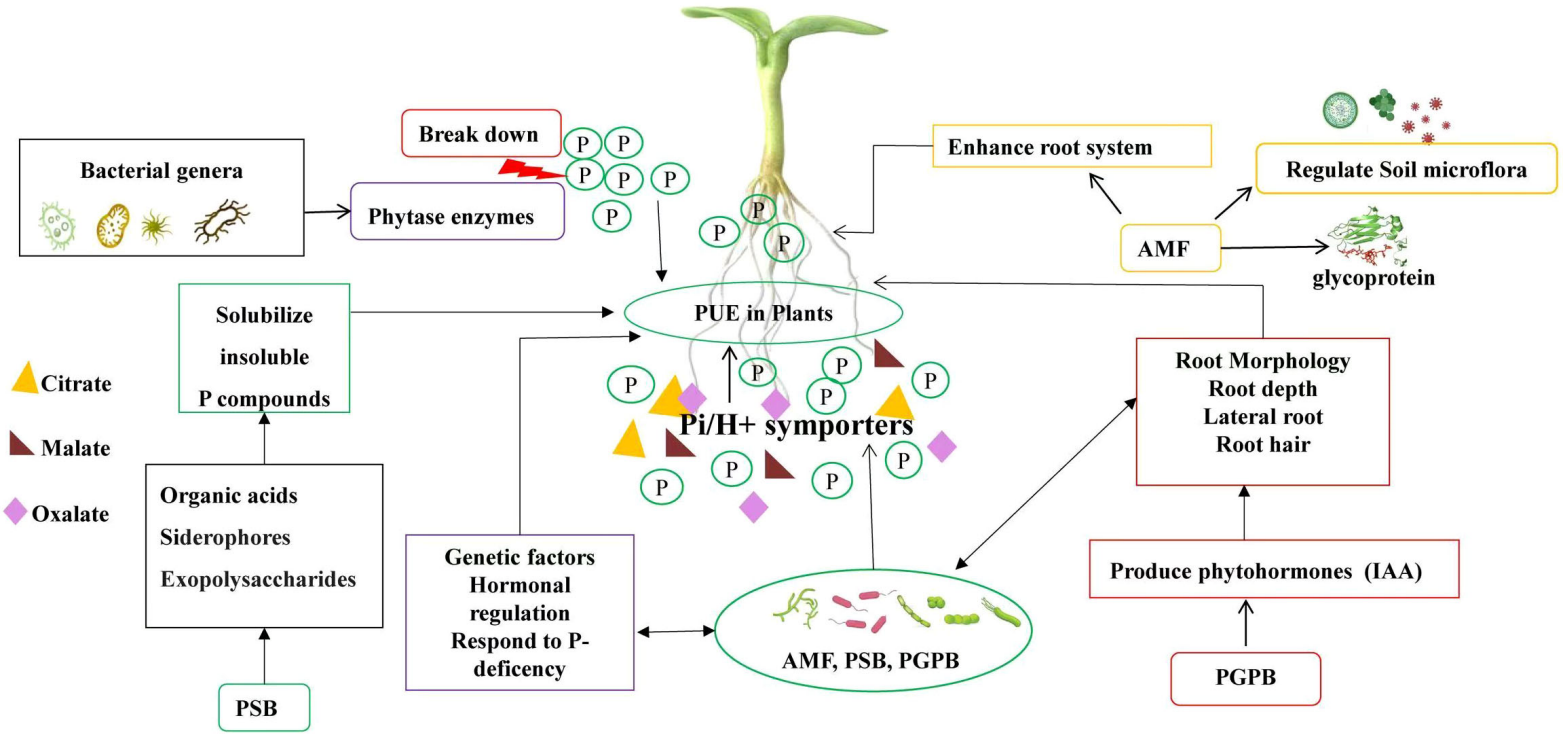
The key components affecting PUE in plants. The central concept, (PUE in Plants), is influenced by three primary factors: (Genetic Factors), which encompass the traits that enhance Pi-uptake and utilization; (Root Morphology), which refers to the structural adaptations of roots that facilitate P absorption; and (Rhizosphere Microorganisms), highlighting the role of microbial communities in enhancing nutrient availability.

Since, P is not only a fundamental macronutrient for plants, but its limited availability in soils poses significant challenges for agricultural productivity, especially under Low-P conditions. This subsection explores the specific factors influencing PUE in plant roots, including root morphology, genetic traits, and the vital roles played by rhizosphere microorganisms such as arbuscular mycorrhizal fungi (AMF), phosphate-solubilizing bacteria (PSB), and plant growth-promoting bacteria (PGPB) (**Figure 3**).

### 4.1 Root morphological adaptations to low-P conditions

The adaptation of plant root morphology to low P conditions is strongly influenced by crop genotypes and regulated by both plant regulators and inherent genetic factors. P acquisition in different crop genotypes relies on the development of an extensive root system that grows in multiple directions until it encounters areas with available P. The growth pattern of the primary root (radicle) plays a crucial role in shaping the overall root morphological structure as it represents the initial point of root growth. This growth pattern is determined by the genetics of the specific crop, which control the processes of cell proliferation and differentiation within the primary root’s meristematic cells (Sánchez-Calderón et al., 2006; Desvoyes et al., 2021). Under low P conditions, plants respond by undergoing various changes in root morphology (**Figure 3**). For example, common beans experience inhibition of root secondary growth and the formation of more root cortical aerenchyma, along with a reduction in lateral root numbers and an increase in primary root formation and whole root depth. Similarly, maize exhibits an increase in primary root formation and whole root depth (Li et al., 2012). Rice, on the other hand, shows a reduction in lateral root length (Vejchasarn et al., 2016), while sugarcane exhibits a shallower root distribution. Conversely, soybean and Arabidopsis show an increase in primary root length, number of lateral roots, and root hair density (Bello, 2021). Additionally, these plants also form root clusters, which can alter the root structure and result in reduced plant growth and an increased root-to-shoot ratio (Strock et al., 2018). These changes in root morphology have been found to enhance plant efficiency under low P conditions.

A recent study found that root hairs play a significant role in acquiring P by increasing the proximity between soil P pools and the root surface (Chen et al., 2021; Liu, 2021). This is particularly important for seedlings that rely on root uptake when stored P in seeds is depleted under low P soil conditions. It also results in less disturbance to root growth compared to the shoot, leading to an increased root-to-shoot ratio.

### 4.2 Microbial interactions and P availability

The synergistic association between plant roots and rhizosphere microorganisms, particularly AMF, is pivotal for enhancing P uptake (Huo et al., 2022). These fungi are endomycorrhizal organisms that penetrate the cell walls of roots and connect with the plasmalemma, allowing the plants to access P pools beyond the reach of roots. Fungi extend their hyphae into the inner cortex of roots, providing a highly efficient interface for signaling and nutrient exchange (**Figure 2**). This symbiosis not only improves nutrient absorption but also stimulates root growth by increasing lateral root length and overall root biomass, especially under P-deficient conditions.

Moreover, AMF release glomalin, a glycoprotein that facilitates the uptake of nutrients like P and iron that are often hard to dissolve (Smith and Read, 2010). Recent research conducted by Zhang et al. (2019) demonstrated that *Zenia insignis* seedlings inoculated with three AMF species, *Funneliformis mosseae, Rhizoglomus intraradices*, and *Diversispora versiformis*, significantly enhanced their growth and drought tolerance (Zhang et al., 2019). Although the association between AMF and plants is generally beneficial for plant growth, it is not always advantageous due to the parasitic nature of the fungi, which can incur carbon costs for the plants. Nevertheless, research has shown that 70-90% of plant species, including ferns, bryophytes, flowering plants, and most agricultural crops, are capable of forming interdependent connections with AMF (Sinha and Tandon, 2020).

Additionally PSB and PGPB (Gupta et al., 2021) or Plant growth-promoting rhizobacteria (PGPR) (Chea et al., 2021) also play a crucial role in the biogeochemical cycling of organic and inorganic P in the rhizosphere. These P solubilizing bacteria enhance P availability for plants by secreting organic acids, siderophores, exopolysaccharides that solubilize insoluble P compounds (**Figure 3**). They produce enzymes like phytases that break down organic P, releasing inorganic forms for plant uptake. Furthermore, PSB form biofilms around roots, improving moisture retention and root health.

Meanwhile, PGPB produce phytohormones (e.g., indole-3-acetic acid, or IAA) that stimulate root growth and lateral root formation (**Figure 3**). Studies indicate that PGPB or PGPR enhance organic P availability, particularly inositol phosphates, and improve plants’ access to P (Menéndez and Paço, 2020). for example, under P deficiency, PGPR have been shown to significantly improve root and shoot biomass, root length, and surface area by 32-45% in potatoes (Unno et al., 2005, Richardson and Simpson, 2011; Hyder et al., 2023). Many PGPR can also produce secondary metabolites and phytohormones that can stimulate the hormonal pathways of plants involved in root development. These positive effects of PGPR have been reported in pea and other vegetable crops (Jiang et al., 2012).

Various bacterial genera, such as *Bacillus, Burkholderia, Enterobacter, Pseudomonas, Serratia*, and *Staphylococcus*, produce phytase enzymes that break down phytate and release P for plant uptake (**Figure 3**). However, the effectiveness of rhizobacteria in solubilizing P can vary depending on soil types, nutrient concentrations, and crop types. It’s important to note that the synergy between different bacterial species and nutrient interactions in the soil can enhance P solubilization and mineralization efficiency.

### 4.3 Mechanisms of action: enzymatic and genetic interactions

Plants under P deficiency respond by, secreting organic acids, (mainly citrate, malate and oxalate), into the rhizosphere, to enhance the availability of inorganic P in the soil (Pang et al., 2015) (**Figure 3**). The quantity and quality of root exudates can vary under different environmental stress conditions. In a P-deficient environment, plants produce and secrete more organic acids, which helps destabilize organic matter and promote the cycling of organic P and P into the soil solutions (Tiziani et al., 2020). Crop genotypes that exhibit a higher ability to exude organic acids in response to low soil P availability can enhance plant-available P and improve PUE in growing plants.

In terms of nutrient uptake, plants exhibit an increased efficiency through high-affinity Pi/H+ symporters (belong to the PHT1 gene family) (**Figure 3**), associated with plasma membranes, which facilitate the uptake of rhizospheric P (Gu et al., 2016). Additionally, plants also induce enzymes that scavenge and recycle Pi, such as acid phosphatase that hydrolyzes Pi from Pi–monoesters, nuclease that degrades extracellular DNA and RNA, and phosphodiesterase that liberates Pi from nucleic acids (Gaume et al., 2001).

### 4.4 Integration of microbial and genetic approaches for improved P efficiency

Exploring the interactions between microbial communities, such as AMF and PSB like *Flavobacterium* C2, along with the inherent genetic variations across different crop genotypes, is essential for optimizing PUE in Low-P environments. Previous studies show that maize plants inoculated with AMF have improved root development and P-uptake compared to non-mycorrhizal plants (Etesami, 2020). In a similar vein, *Flavobacterium* C2 enhances P availability through the production of organic acids, such as citric and malic acids, which can lower rhizosphere pH and increase the solubility of mineral P (Liu et al., 2024).

This highlights how beneficial microorganisms can play a vital role alongside genetic traits tailored to improve P acquisition. By integrating insights from both genetics and microbial ecology, agricultural practices can be significantly enhanced to improve crop resilience and productivity. Focusing on breeding programs that enhance root traits—like increased root hair density or deeper rooting systems—while also applying beneficial microbes like C2 provides critical pathways to enhance P-uptake. Additionally, C2’s ability to produce IAA may further stimulate root growth, supporting plants in P-deficient soils. This comprehensive strategy not only optimizes plant growth but also encourages sustainable agricultural practices that are crucial for addressing global food security challenges.

## 5 Impact of phosphorus on respiratory metabolism and ATP production under low-P conditions

Plant respiratory metabolism breaks down glucose and other organic molecules to produce energy in the form of ATP. P plays a crucial role in this process by synthesizing and activating key molecules involved in respiratory metabolism, including ATP. This involves a series of enzymatic reactions in the mitochondria, starting with the breakdown of glucose via glycolysis and subsequent oxidation in the tricarboxylic acid cycle. The final stage, oxidative phosphorylation, occurs in the electron transport chain, where P is used to synthesize ATP from ADP and Pi (Deshpande and Mohiuddin, 2020). During long-term Pi stress, the decline in adenylate and Pi content restricts the activity of several enzymes involved in classical glycolysis and mitochondrial respiration that depend on ATP, ADP, and/or Pi. However, the cytosolic level of inorganic pyrophosphate (PPi) remains relatively stable. PPi, a byproduct of biosynthetic reactions, plays a crucial role in enhancing cellular processes’ energetic efficiency (Dissanayaka et al., 2021). Interestingly, PPi-dependent reactions are utilized by anaerobic microorganisms and plant cells to yield ATP and recycle Pi. This suggests that the upregulation of PPi-dependent enzymes in -Pi plant cells may play a significant role in respiration and Pi recycling during Pi stress. It connects the importance of P not only in ATP production but also in overall metabolic adaptation under Pi limitations.

## 6 Epigenetic modulation of phosphorus acquisition and utilization

Epigenetic modifications, including DNA methylation and histone modifications, miRNA, and LncRNA, play a crucial role in regulating various plant processes, including the uptake and utilization of P. By influencing gene expression, these modifications impact nutrient acquisition and metabolism (Li et al., 2021). Notably, the addition of a methyl group to DNA molecules can either enhance or suppress key genes involved in P pathways, ultimately affecting a plant’s ability to efficiently acquire and utilize P. For instance, in Arabidopsis, DNA methylation levels increase in response to Pi deficiency, leading to changes in root growth and density of root hairs under Pi-replete conditions. This suggests that regulatory components involved in the P starvation response, such as SPX2 and miR827, experience differential methylation (Yong-Villalobos et al., 2015). Moreover, DNA methylation selectively modulates Pi signaling through important cis-elements like P1BS, WRKY, and MYB motifs (Yong-Villalobos et al., 2016). The identification of specific regulatory components like SPX2 and miR827 reveals potential targets for genetic intervention. Manipulating their expression could enhance plants’ adaptive responses to P deficiency, thereby fostering more sustainable agricultural practices and improving crop yields in P-limited soils. In maize and rice, the response to P deficiency in terms of DNA methylation may differ (Li et al., 2021). For instance, in maize, Low-P conditions have only a minor effect on global DNA methylation levels. In contrast, long-term P deficiency in rice leads to gradual changes in gene expression and DNA methylation patterns, which persist even after P supply is restored (Secco et al., 2015).

Recently Gladman et al. (2022) found that sorghum responds to Low-P conditions by showing a decrease in global DNA 5-methylcytosine and H3K4 and H3K27 trimethylation levels. Interestingly, they discovered that H3K4me3 peaks and DNA hypomethylated regions contain regulatory motifs for various developmental and nutrient-responsive transcription factors, including SHORTROOT (SHR), SCARECROW (SCR), and ROOTLESS CONCERNING CROWN and SEMINAL ROOTS (RTCS). Additionally, these regions exhibit distinct expression patterns among different root tissues, such as the primary root apex, elongation zone, and lateral root apex (Gladman et al., 2022). The response to DNA methylation under P deficiency, as well as the specific genes involved, can vary across different species (Gladman et al., 2022).

Histone modifications have a pivotal role in regulating gene expression associated with P metabolism. These modifications impact the accessibility of genes to regulatory proteins and transcription factors. Acetylation of histones, in particular, promotes gene expression by relaxing chromatin structure and facilitating the binding of activating proteins. One gene involved in histone acetylation, *AtGCN5*, plays diverse roles in plant development and stress response (Xing et al., 2015; Zheng et al., 2019). Mutation of *AtGCN5* affects P accumulation and impairs the activation of genes like *At4* and *AtWRKY6* under low-P conditions (Wang et al., 2019). In addition to acetylation, methylation is another important histone modification that influences gene expression in P metabolism. Methylation at specific loci can recruit activating proteins or modify chromatin structure, thereby favoring gene transcription. This modification can promote the expression of P metabolism-related genes by facilitating the binding of transcription factors or remodeling the chromatin architecture. For instance, in Arabidopsis, the ALFN protein encoded by *AtAL6* plays a role in root hair elongation under low-P conditions (Chandrika et al., 2013). *AtAL6* recognizes H3K4me3, a specific form of histone methylation, through its Plant Homeo Domain (PHD) finger. By recognizing H3K4me3, *AtAL6* promotes the transcription of *AtETC1*, a gene involved in root growth, and activates downstream targets such as *AtNPC4, AtSQD2*, and *AtPS2* (Taverna et al., 2006; Wei et al., 2017). This ultimately leads to root hair elongation in response to P deficiency. Therefore, histone methylation, particularly the recognition of specific methyl marks by proteins like *AtAL6*, is crucial for regulating the expression of P metabolism-related genes (Chandrika et al., 2013).

Conversely, histone modifications can also act as repressive signals. Methylation of histones at specific lysine residues, like H3K9, is often associated with gene silencing. In the case of P metabolism-related genes, methylation at repressive loci can recruit proteins that inhibit gene expression or induce a more compact chromatin structure, thereby preventing the access of activating proteins. Similarly, the deacetylation of histones is linked to gene repression. Deacetylated histones result in a condensed chromatin structure, which is typically associated with gene silencing. Deacetylation at the promoter regions of P metabolism-related genes can suppress their expression by limiting the accessibility of transcription factors and other activating proteins. In addition to histone modifications and DNA methylation, there are some other epigenetic regulations such as substitution of histone variant H2A.Z, nucleosome remodeling, and chromatin accessibility, which can also modulate gene expression (Zovkic et al., 2014).

Furthermore, Post-transcriptional regulation by miRNAs and LncRNAs adds an epigenetic control layer to gene expression. These non-coding RNAs bind to mRNA, leading to degradation or translation inhibition. They have a significant impact on plant gene regulation, generating phasiRNAs that influence plant development, physiology, and stress response (Meng et al., 2021). In maize, miR399 regulates *ZmPHO2* expression, maintaining P homeostasis by targeting it for degradation and controlling genes involved in P uptake and utilization (Du et al., 2018). While Two lncRNAs, *At4* and *IPS1*, are induced by Pi starvation in Arabidopsis. These lncRNAs have orthologous counterparts in Medicago (*Mt4*) and tomato (*TPS1*) (Shin et al., 2006). Mutations in *At4* lead to problems in Pi redistribution between the root and shoot under deficient conditions. The members of the *At4/IPS1* gene family contain a conserved motif that is partially complementary to miR399. However, a central mismatch prevents cleavage of the transcript by miR399 (Huang et al., 2013). As a result, the binding of *At4/IPS1* lncRNAs acts as a decoy, reducing miR399 activity and consequently upregulating its target transcript, PHO2. PHO2 encodes the ubiquitin-conjugating enzyme UBC24, which mediates the degradation of several high-affinity PHT1 and the PHO1 protein involved in Pi loading in the xylem (Huang et al., 2013). Recently Yuan et al. (2016), used a genome-wide sequencing approach to identify lncRNAs responsive to P starvation in Arabidopsis. They predicted 1212 novel lncRNAs, including 78 poly(A)- lncRNAs, some of which were associated with genes involved in Pi starvation-related processes. Furthermore, they identified 104 lncRNAs as potential targets of PHR1 and 16 lncRNAs as potential targets of miR399, both important regulators of plant Pi homeostasis (Yuan et al., 2016). Similarly, Zhang et al. (2021a) conducted RNA sequencing on soybean roots of different genotypes with varying P tolerance. They identified 4,166 novel lncRNAs, of which 525 were differentially expressed under different P levels. These differentially expressed lncRNAs were found to be associated with various Pi-related biological processes based on GO and KEGG analysis (Zhang et al., 2021b). Additional Computational analysis of Arabidopsis and rice genomes revealed numerous potential lncRNAs that mimic miRNA targets. The role of *miR160* and *miR166* target mimics in Arabidopsis development was confirmed. This suggests that lncRNAs have a general regulatory mechanism for controlling miRNA activity in plants (Wu et al., 2013).

Recent studies have also implicated circular RNAs (CircRNAs) in the epigenetic regulation of P metabolism. CircRNAs, a type of non-coding RNA, are formed through a head-to-tail splicing process, resulting in a covalently closed loop structure. Emerging evidence suggests that CircRNAs can modulate various plant processes, including P acquisition and utilization (Pan et al., 2018). For instance, novel_circ_000013, novel_circ_000349, novel_circ_000351, and novel_circ_000277 have been found to interact with multiple miRNAs (Lv et al., 2020). One mechanism by which CircRNAs regulate P metabolism is through their role as miRNA sponges. CircRNAs bind to miRNAs, preventing their interaction with target mRNAs and effectively sequestering them (Zhou et al., 2020). This sequestration of miRNAs by CircRNAs, where miR399, miR319, miR156 and miR159 were sponged, lead to the upregulation of key genes involved in P pathways. Additionally, CircRNAs directly interact with proteins and influence their activity, thereby impacting P acquisition and utilization (Lv et al., 2020). However, further research is needed to fully understand the specific mechanisms by which CircRNAs function in the context of P metabolism and their potential roles in plant adaptation to P deficiency.

## 7 Conclusion and future perspectives

Improving crop PUE is critical for minimizing P fertilizer use, protecting the environment from eutrophication, and conserving global P mineral resources (Bello, 2021). Historical analyses show that global PUE has significantly improved, rising from 44% in the 1980s to approximately 66% in 2019, despite an increase in P fertilizer usage. This improvement highlights the urgent need to address challenges posed by P pollution and diminishing rock reserves.

Recent studies, including GWAS by Rajamanickam et al. (2024), identified 14 PUE traits in wheat, revealing substantial SNP associations and significant advancements in understanding P transport through the candidate gene *TaPHT1;9* (Rajamanickam et al., 2024). Moreover, functional studies on CRISPR-edited mutants in transgenic rice demonstrated notable increases in grain yield, biomass, P concentration, and overall PUE under Low-P conditions (Chen et al., 2024). It is evident that plants can reprogram their gene expression in response to P deficiency—engaging various regulatory pathways, particularly involving TFs such as WRKY. For instance, transgenic soybeans overexpressing *GmWRKY46* have shown improved root development, enhancing plant growth and P uptake (Li et al., 2021a). Additionally, genetic contributions to low P tolerance, such as those from the *Gm6PGDH1* gene, indicate pathways to enhance PUE further (Li et al., 2021b). Other P signaling pathways PHO, MYB62 and SPX pathway also occupy a very important position in the P signaling network, which is tightly related to P uptake, transport, storage and homeostasis. Further exploration of their involvement in epigenetic regulation of P can provide valuable insights (**Figure 1**). The analysis of Arabidopsis PHO1 (*AtPHO1*) with its EXS domain alongside SPX (Ribot et al., 2008) offers a potential illustration of how these pathways may contribute to the interplay between P, enzymes, and phytohormone signaling pathways to improve PUE.

The interplay between plant roots and rhizosphere microorganisms is vital for enhancing PUE in Low-P environments. For example, the fungi *Funneliformis mosseae* play a significant role in improving P content and promoting growth in rice through systemic gene expression changes (Campo and San Segundo, 2020). Moving forward, future research should focus on integrating microbial and genetic approaches to enhance PUE. Identifying key genetic traits that facilitate beneficial microbial interactions and exploring multi-strain microbial inoculations could further optimize P availability. Ultimately, harnessing these microbial relationships can lead to more sustainable agricultural practices, reducing reliance on chemical fertilizers while improving soil health and crop resilience.

Advancements in PUE research are expected to significantly influence plant physiology and breeding strategies. However, a limited understanding of the genetic basis underlying PUE continues to hinder the development of P-efficient cultivars (van de Wiel et al., 2016).

Recent advancements in crop genomics, including SNP marker development and pan-genome assemblies, have made identifying genomic regions associated with PUE more efficient. Resources like genomic selection and prediction methods can facilitate the creation of progenies with improved PUE. The implementation of speed breeding protocols will further streamline breeding timelines for mapping populations. Furthermore, high-throughput phenotyping innovations are crucial for advancing our understanding of root morphology and architecture in relation to P availability. Techniques such as 3D laser scanning and digital imaging facilitate robust assessments of root traits in variable environments (Fang et al., 2009). Additionally, integrating modern technologies like machine learning and genome editing can markedly improve breeding strategies.

In conclusion, a multifaceted approach that incorporates genetic, molecular, physiological, and epigenetic strategies is essential for developing crops capable of thriving in Low-P conditions. Such advancements will be instrumental in addressing global food security challenges in the face of climate change and resource depletion.
